# Composite Membranes Containing Nanoparticles of Inorganic Ion Exchangers for Electrodialytic Desalination of Glycerol

**DOI:** 10.1186/s11671-017-2208-4

**Published:** 2017-06-30

**Authors:** Yu S. Dzyazko, L. M. Rozhdestvenska, S. L. Vasilyuk, K. O. Kudelko, V. N. Belyakov

**Affiliations:** 0000 0004 0385 8977 grid.418751.e V.I. Vernadskii Institute of General & Inorganic Chemistry of the NAS of Ukraine, Palladin Ave. 32/34, 03680 Kiev 142, Ukraine

**Keywords:** Organic-inorganic membranes, Electrodialysis, Glycerol, Hydrated zirconium dioxide, Zirconium phosphate

## Abstract

Composite membranes were obtained by modification of heterogeneous polymer cation and anion-exchange membranes with nanoparticles of zirconium hydrophosphate and hydrated zirconium dioxide, respectively. The ion-exchange materials were investigated with the methods of electron microscopy, potentiometry, voltammetry, and impedance spectroscopy. Single nanoparticles, which were precipitated in aqueous media, form aggregates, when the composites are in a contact with polar organic solvent. Both single nanoparticles (up to 10 nm) and their aggregates (up to 200 nm) were precipitated in ion-exchange polymers in glycerol media. Non-aggregated nanoparticles improve electrical conductivity of the ion-exchange materials, the aggregates are barriers against fouling. The membranes were applied to NaCl removal from highly concentrated glycerine-water mixture containing organic additives (byproduct of biodiesel production). As opposite to pristine materials, the composites demonstrate stability against fouling.

## Background

Electrodialysis is applied as a solution of different problems: water treatment and water conditioning [1], particularly removal of toxic ionic components from ground water [2–4] or preparation of water from liquid wastes of dairy industry for washing of equipment [5], processing of reverse osmosis concentrate [6], or secondary wastes after sorbent regeneration [7], desalination of protein concentrate [8], production of organic acids [9], and many other practical tasks.

Very important practical problem is processing of non-aqueous solutions, for instance, glycerol, which is formed as a byproduct during biodiesel production [10]. Glycerol can be further used for synthesis of dihydroxyacetone, succinic, propionic, citric acid, pigments, etc. [11], for production of synthetic gas [12] and even as fuel [13]. However, preliminary deep desalination is necessary since glycerol produced by this manner contains high amount of mineral components (mainly NaCl). The most common purification method is extremely energy-intensive distillation [14]. Ion exchange [15] as well as reverse osmosis [16] could be applied only to slightly mineralized solution. Ultrafiltration, which has been proposed for removal of palm and oleic acid from glycerol [17], cannot be applied to desalination.

Electrodialysis is expected to be the most suitable method for glycerol desalination since the process can be used for removal of inorganic ions from solutions of wide concentration interval [18, 19]. Bipolar electrodialysis has been developed earlier for glycerol desalination: the demineralization degree above 80% was achieved with glycerol losses below 2% [18]. Traditionally, polymer ion-exchange membranes are used for electrodialysis [20]. In the case of crude glycerol, which contains high amount of organic additives, fouling of the polymer membranes is expected [21–23].

In the case of materials for baromembrane separation, modification of the membranes with inorganic nanoparticles (SiO_2_ [24], Fe_2_O_3_ [25], ZrO_2_ [26, 27], TiO_2_ [28], zirconium hydrophosphate [29]) provides their stability against fouling with organics. Similar approach was applied to ion-exchange membranes for fuel cells [30–32]. Functions of the inorganic modifier are to enhance proton conductivity of the membranes and to prevent their dehydration under high temperature. Last years, the organic-inorganic membranes for electrodialysis were investigated [33–37]. Nanoparticles of inorganic ion exchanger transform even inert polymer to ion-exchange membrane [35], it is similarly to ceramic membranes [38–40]. However, polymer ion-exchange membranes are poisoned with organic solvents [41, 42]: reorganization of their porous structure results in deterioration of functional properties, for instance, it enhances methanol crossover [42]. This undoubtedly influences location of inorganic particles, which have to be non-aggregated in order to provide high rate of ion transport [43, 44].

The aim of the work was to obtain organic-inorganic membranes for desalination of non-aqueous solutions, which would combine stable structure in these media, high charge selectivity, considerable electric conductivity, and stability against fouling with organics. The task of the work is the development of the modification methods using ion-exchange resins as model polymer matrices since these materials are used for preparation of heterogeneous membranes. Other problems are the application of the modification technique to membrane preparation, the investigation of morphology and functional properties of the composite materials, the testing of the membranes in the process of desalination of crude glycerol.

Hydrated zirconium dioxide (HZD) was used as a modifier of anion-exchange membrane. This ion-exchanger demonstrates anion-exchange ability in acidic and neutral media [45]. Amorphous zirconium hydrophosphate (ZHP) was applied to modification of cation-exchange membranes. This inorganic ion exchanger possesses high exchange capacity, it is chemically stable and requires no expensive chemical reagents for synthesis.

## Experimental

### Solutions for Electrodialysis

The effluent obtained during biodiesel production (Trostyanetz distillery plant of "Ukrspirt State Comrany", Ukraine) was applied to investigations. This glycerol-based solution contained water (10 mass %), organic impurities (8 mass %), and 1000 mol m^−3^ NaCl. Aqueous NaCl solutions were also used for potentiometric and impedance measurements.

### Modification of Ion-Exchange Resins

Granulated polystyrene-divinylbenzene gel-like resins, namely, Dowex HCR-S (strongly acidic cation exchanger) and Dowex Marathon A (strongly basic anion exchanger), which had been produced by Dow Chemical company, were researched preliminary. It was necessary for investigations of the composites with transmission electronic microscopy (TEM) and for a choice of the most suitable modification method. The cation exchanger and anion exchanger were modified with ZHP and HZD, respectively.

The first series of the samples was prepared in accordance with following stages: (i) impregnation of the resin with water, (ii) impregnation of the wet resin with a 1 M ZrOCl_2_ solution for 24 h at 298 K (a ratio of volumes of the resin and solution was 1:20), (iii) washing of the resin with a HCl solution (10 mol m^−3^) up to constant pH of the effluent (about 2) to remove additionally sorbed electrolyte as completely as possible, (iv) treatment of the resin with a 1 M H_3_PO_4_ solution at 298 K (a ratio of volumes of the resin and solution was 1:10) followed by washing with deionized water up to neutral reaction of the effluent, (v) treatment with ultrasound at 30 kHz by means of a *Bandelin* device (*Bandelin*, Hungary) in order to clean outer surface of the granules, and (vi) treatment with glycerol followed by washing with deionized water and drying in a desiccator over CaCl_2_ at room temperature down to constant mass. After stage (v), a part of the resin was taken and dried in the desiccator.

Regarding the anion exchanger, the modification procedure was similar. However, a mixed solution (1 M ZrOCl_2_ and 7 M HCl) was used for resin impregnation (stage ii), 7 M HCl was employed for washing until disappearance of turbidity of the effluent after neutralization (stage iii). The inorganic constituent was precipitated with a 1 M NH_4_OH solution (stage iv).

The second series of the samples was prepared similarly; however, a 0.1 M ZrOCl_2_ solution in glycerol was used for resin impregnation. Solutions of H_3_PO_4_ or NH_4_OH in glycerol were used for precipitation of ZHP or HZD, respectively.

### Modification of Ion-Exchange Membranes

CMI 7000 cation exchange (CM) and AMI 7000 anion exchange (AM) heterogeneous membranes (*Membrane International*), a thickness of which in a swelling state is about 600 μm, were investigated. The membranes were modified with HZP and HZD, respectively. The modification procedure was similar to that described above for the second series of the samples. After the last drying, the membranes were weighted.

### SEM and TEM

Investigations of the membranes with a method of scanning electron microscopy (SEM) were provided by means of *JEOL JSM 6700 F* and *JEOL JFC-1600* microscopes (*JEOL,* Japan). Preliminary, a platinum layer was deposited onto the sample at 3 Pa using an JEOL JFC-1600 Auto fine coater (JEOL, Japan). A *JEOL JEM 1230* transmission electron microscope (JEOL, Japan) was applied to crushed ion-exchange resins. Before the investigations, both the membranes and resins were treated with ultrasound.

### Investigation of Ion Transport

Two-compartment divided cell supplied by Ag/AgCl electrodes was used for potentiometric measurements, which were performed by means of a *SCH-1312* voltmeter (Analitpribor, Ukraine). The cell compartments were filled with aqueous NaCl solutions (0.5 and 1 M) similarly to [46, 47].

Electrical resistance of the membranes was measured using a two-compartment cell supplied with platinum electrodes. Aqueous NaCl solutions filled the cell. The measurements were performed using an Autolab impedance system at 1 × 10^−2^ − 1 × 10^6^ Hz. The cell resistance was determined as a wide plateau of frequency dependence of the real part of impedance. The membrane resistance was calculated as a difference between resistances of the cell with and without membrane [47, 48]. For comparison, electrical conductivity of H–(OH) forms of the ion-exchange resins was measured similarly to [43, 44]. Deionized water was used as a non-conducting medium.

Voltammetric measurements were provided according to four-electrode scheme similarly to [46]. The scheme involved two-compartment divided cell, two platinum working electrodes, which were connected with a *IPPT 65-49* power supplier (*Ukrrospribor LTD*, Ukraine) and a *SCH-4311* ammeter (*Analitprobor*, Ukraine). Two Ag/AgCl electrodes were connected with a voltmeter. The reference electrodes were supplied with Luggin capillaries.

All experiments were carried out at 298 K.

### Electrodialysis of Glycerol Solution

**Fig. 1:**
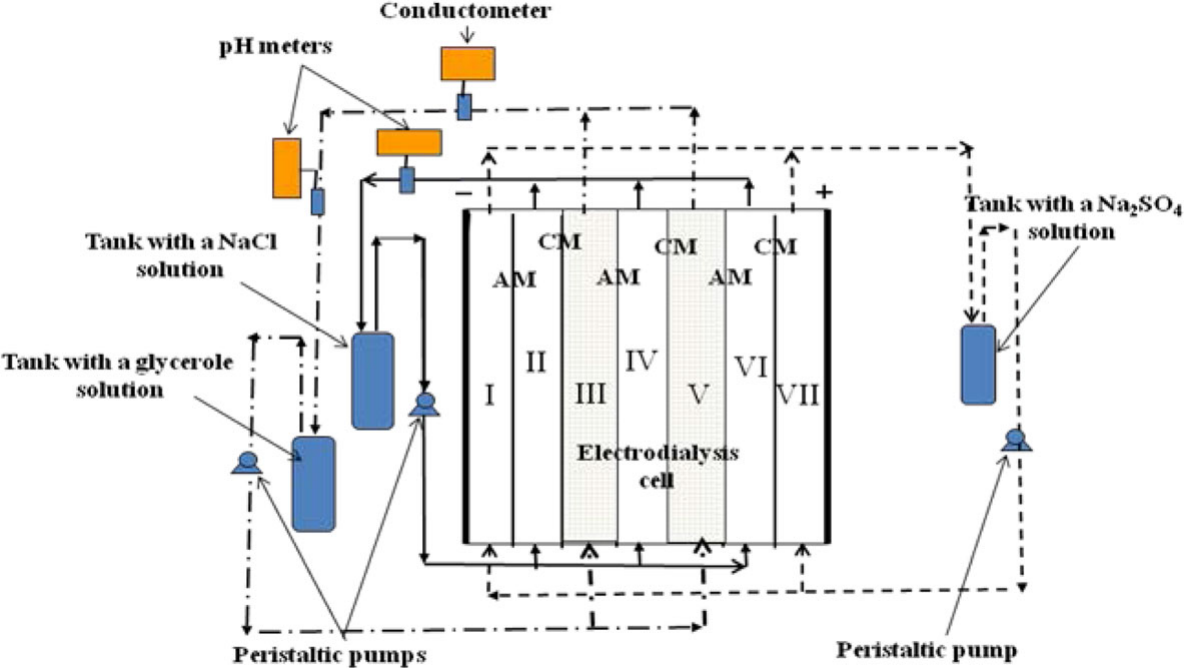
Experimental setup for glycerol electrodialysis. Sections *I* and *VII* are the electrode compartments; *II*, *IV*, and *VI* are the concentration compartments; *III* and *V* are the desalination compartments

Experimental setup involved seven-compartment cell, three independent liquid lines, power supplier, and measuring instrumentation mentioned above (Fig. 1). The desalination chambers contained a grid for flow turbulization. An effective membrane area was 30 cm^2^ (30 cm × 1 cm), a distance between the membranes was 4 mm, a cross section area of each compartment was 0.4 cm^2^. The composite membranes were placed between the desalination and concentration compartments, other membranes were pristine. For comparison, the separation process was performed using only pristine membranes between all compartments.

A glycerol solution (200 cm^3^) was passed through the desalination compartments according to cyclic operation. A NaCl solution, initial concentration of which was 0.01 M (200 dm^3^) circulated through the concentration compartments. A 0.05 M Na_2_SO_4_ solution was passed through the electrode compartments.

## Results

### Aggregation of Nanoparticles Inside Polymer Matrix

Location of inorganic particles inside polymer ion-exchangers is determined by porous structure of these materials in a swollen state. The structure is known to include gel-like regions, where nanosized clusters (up to 20 nm [36, 43, 44, 46, 49–51]) and narrower channels between them are located (cluster-channel structure of polymer ion-exchange materials is described in detail in [49–51]). Clusters and channels, which contain functional groups, are considered as transport pores. Hydrophobic fields of hydrocarbonaceous chains are placed in voids between gel fields. Pores of micron size are related to structure defects and voids between ion exchanger and binder (for heterogeneous membranes).

**Fig. 2:**
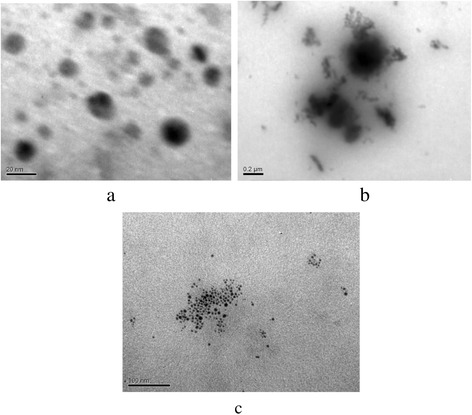
TEM images of ZHP (**a**, **b**) and HZD **c** nanoparticles in the cation (**a**, **b**) and anion (**c**) exchangers of series 1 before (**a**, **c**) and after (**b**) treatment with glycerol. Non-aggregated nanoparticles (**a**, **c**) and their aggregates (**b**) are seen

Visualization of non-aggregated inorganic nanoparticles is possible only for resins: their grains can be crushed relatively easy down to size, which allows us to obtain TEM images. The photos of the organic-inorganic cation exchanger of the first series before and after treatment with glycerol (i.e., after modification stages v and vi) are given in Fig. 2. Non-aggregated globular ZHP nanoparticles (4–20 nm) can be seen, the aggregates were found to be practically absent. Non-aggregated HZD nanoparticles were also found in the anion exchanger. The nanoparticles are evidently placed inside clusters and channels and stabilized by their walls.

After treatment with organic solvent, no single nanoparticles were found. They form aggregates (≈100 nm), which are evidently located outside transport pores. The aggregation is probably due to cluster reorganization caused by adsorption of organic solvent [42]. Moreover, the reorganization can be caused by lower dielectric permittivity of glycerol in comparison with water. This enhances repulsion of counter ions of functional groups. As a result of reorganization, the nanoparticles leave the transport pores and form aggregates outside them.

**Fig. 3:**
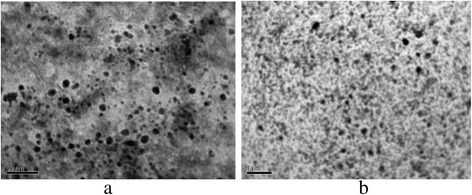
TEM images of cation - exchanger of the second series. ZHP nanoparticles (**a**) and larger particles, a size of which is 100–300 nm (**b**), are visible

Non-aggregated ZHP nanoparticles (2–10 nm), which were precipitated from glycerol solution, are seen in the sample of the second series (Fig. 3). Larger particles (up to 300 nm) are also visible on the image with smaller resolution. These particles are evidently related to aggregates, which are evidently places in voids between gel regions.

**Fig. 4:**
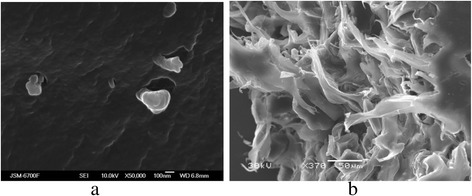
SEM images of cross section of composite cation-exchange membrane. Small aggregates of ZHP nanoparticles are seen (**a**), no particles are seen in large pores (**b**)

Similar regularities of nanoparticle formation are evidently characteristic for the membranes. As found, the mass content of ZHP and HZD in the membranes was 4.5 and 3.9%, respectively. After the treatment with glycerol, small aggregates (up to 300 nm) were found inside the ion-exchange constituent of the membranes (Fig. 4). These aggregates are evidently located in the voids between gel regions. No large particles, a size of which is comparable with pores between the ion-exchange polymer and binder, were found (Fig. 4).

### Electrical Conductivity and Charge Selectivity of the Membranes

**Fig. 5:**
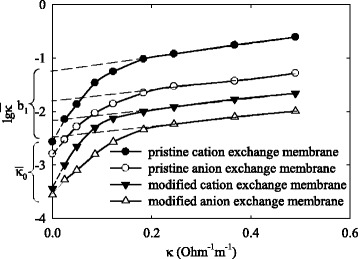
Logarithm of membrane conductivity vs conductivity of outer aqueous NaCl solution

Logarithm of specific electrical conductivity of the membranes $\left( \log \overline{\kappa}\right)$ is plotted in Fig. 5 vs conductivity of aqueous NaCl solution (*κ*). As seen, a reducing of the solution concentration causes a decrease of the $\overline{\kappa}$ values due to diminution of a content of additionally sorbed electrolyte (both counter and co-ions). The electrolyte fills pores, which contain no functional groups. The $\overline{\kappa}$ magnitude involves ion transport through clusters and channels. When the diffusion parts of electric double layers are not overlapped, this transport is due to surface and fluid conductivity.

In the case of cation-exchange membrane impregnated with a solution, its conductivity is determined as follows:

1 $$ \overline{\kappa}= F\left({z}_{+}{\overline{u}}_{+}^{/}{\overline{C}}_{+}^{/}+{z}_{+}{\overline{u}}_{+}^{//}{\overline{C}}^{//}+{z}_{-}{\overline{u}}_{-}^{//}{\overline{C}}^{//}\right). $$

Here, *F* is the Faraday constant, *z* is the charge number, *ū* is the mobility and $\overline{C}$ is the concentration, “+” and “−” subscripts correspond to cations and anions, respectively, “^/^” superscript is related to counter ions in clusters and channels, “^//^” index is attributed to counter and co-ions in pores, which are free from functional groups. Under the conditions of ${z}_{+}{\overline{u}}_{+}^{/}{\overline{C}}_{+}^{/}<<{\overline{C}}^{//}\left({z}_{+}{\overline{u}}_{+}^{//}+{z}_{-}{\overline{u}}_{-}^{//}\right)$, the concentration of species outside clusters and channels can be determined as ${\overline{C}}^{//}=\frac{\overline{\kappa}}{z_{+}{\overline{u}}_{+}^{//}+{z}_{-}{\overline{u}}_{-}^{//}}$. Here ${\overline{u}}_{+}^{//}$ and ${\overline{u}}_{-}^{//}$ are assumed to be equal to mobility of species in outer solution.

**Fig. 6:**
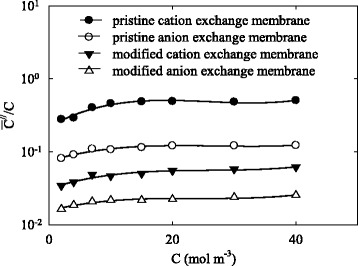
Ratio of $\frac{{\overline{C}}^{.//}}{C}$ as a function of concentration of outer solution

The dependencies of $\frac{{\overline{C}}^{.//}}{C}$ on *C* (where *C* is the concentration of outer solution) are shown in Fig. 6. This ratio increases in the region of low concentration due to depression of surface conductivity through clusters. Further, the ratio reaches approximately constant values. The plateau corresponds to the concentration interval, at which the conductivity is determined mainly by the additionally sorbed electrolyte.

Extrapolation of the curve, which reflects the dependence of $\lg \overline{\kappa}$ on *κ*_,_ to *κ* = 0 gives a rather low magnitude. This value corresponds to ion transport only through the clusters and channels (Table 1). These $\overline{\kappa}$ values are lower for the modified membranes, it is in agreement with data obtained for the ion-exchange resins (Table 2). Linear regions of the curves are related to the concentration diapason, where the conductivity of the membranes is determined mainly by additionally sorbed electrolyte. It is valid for this concentration interval:

**Table 1:** Parameters for characterization of electrical conductivity and charge selectivity of the membranes

Sample	*b*_1_	*b*_2_, Ohm m	$\overline{\kappa}$ × 10^3^ (*κ* → 0), Ohm^−1^ m^−1^	$\overline{t}$
Pristine cation-exchange membrane	−1.25	1.33	2.75	0.97
Pristine anion-exchange membrane	−1.84	1.13	1.62	0.97
Modified cation-exchange membrane	−2.19	1.10	0.29	0.98
Modified anion-exchange membrane	−2.52	1.09	0.25	0.94

**Table 2:** Electrical conductivity of H–(OH) forms of the resins measured in deionized water

Resin	$\overline{\kappa}$, Ohm^−1^ m^−1^
Pristine cation exchanger	0.21
Pristine anion exchanger	0.15
Modified cation exchanger (series 2)	0.11
Modified anion exchanger (series 2)	0.09

2 $$ \lg \overline{\kappa}={b}_1+{b}_2\kappa, $$

where *b*_1_ and *b*_2_ are the empirical coefficients. They reflect the screening effect of polymer matrix (pristine membranes) or both matrix and aggregates (modified membranes).

Lower values of the coefficients for the modified membranes show that the aggregates perform a function of the barrier against additionally sorbed electrolyte. Since organics can be adsorbed on hydrophobic parts of polymer chains, the aggregates are assumed to protect the membranes from fouling.

Transport numbers of counter ions through the membrane ($\overline{t}$) were determined from measurements of membrane potential (*E*_*m*_) followed by calculations from the formula [47]:

3 $$ {E}_m=\left(2\overline{t}-1\right)\frac{RT}{zF} \ln \frac{a_2}{a_1}, $$

where *a*_1_ and *a*_2_ are the activity of less and more concentrated solutions, *R* is the gas constant, and *T* is the temperature.

**Fig. 7:**
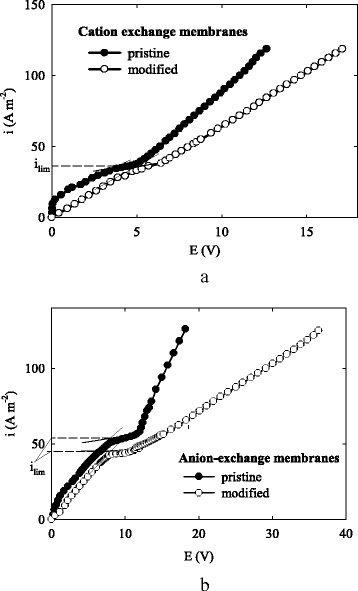
Voltammetric curves for cation-exchange **a** and anion-exchange **b** membranes. The measurements were performed in aqueous solution containing 40 mol m^−3^ NaCl, superficial flow velocity was 1.04 × 10^−3^ m s^−1^

Voltammetric curves obtained for aqueous NaCl solutions according to four-electrode scheme are given in Fig. 7. As seen, the values of limiting current density (*i*_lim_) are practically the same both for the pristine and modified cation-exchange membranes. The modified anion-exchange separator shows slightly lower current density than the pristine membrane indicating deterioration of charge selectivity.

In the region of *i* < 0.75 *i*_lim_, no linearity of the voltammetric dependencies is observed for the pristine membranes. The non-linearity indicates concentration polarization, which occurs in the largest pores between the ion-exchange polymer and binder. This phenomenon is typical for heterogeneous membranes [50]. However, the voltammetric dependence is linear for the modified membranes at *i* < 0.75 *i*_lim_. This indicates an exclusion of ion transport through the largest pores of the composites membranes evidently due to reduction of amount of additionally sorbed electrolyte.

### Desalination of Waste Glycerol

Electrodialysis was performed under the constant voltage, which provided *i* = 0.75*i*_lim_, where *i* and *i*_lim_ are the current density and limiting current, respectively. This was necessary to avoid precipitation of organic additives inside the membrane system. The current gradually decreased in accordance to reduction of NaCl concentration in the desalination compartments. Configuration of the membrane system provided stability of the pH (about 6) both of the concentrate and solution being purified.

**Fig. 8:**
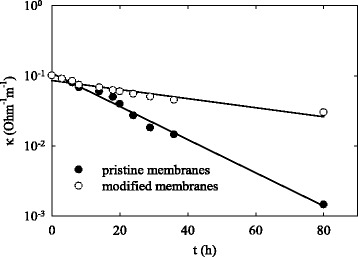
Conductivity of crude glycerol over time of electrodialysis

When the modified membranes separated the desalination and concentration compartments, the salt content in the glycerol solution diminished gradually. This reflects a dependence of electrical conductivity of the solution through the desalination compartment on time plotted in semi-logarithmic coordinates (Fig. 8).

Linear dependence in these coordinates is due to diffusion limitations. No organic impurities, which were present in crude glycerol, were found in the desalination compartment. The current efficiency reached 95–98%. The process was stopped, when the residual salt concentration was in 1000 times lower than that in the initial solution. After finishing the process, the membranes were removed, washed with deionized water, and their conductivity was measured using aqueous NaCl solution (40 mol dm^−3^) as described in “Investigation of Ion Transport.” A decrease of conductivity was about 2% for the cation-exchange membrane in a comparison with the value obtained before the process. Regarding the anion-exchange separator, the conductivity was even slightly higher after electrodialysis (about 5%). However, these deviations are practically within experimental error indicating stability of the modified membranes against fouling.

In the case of the pristine membranes, the rate of desalination is much slower evidently due to their blockage with organics. The cell voltage increased dramatically. Moreover, the solution through the desalination compartment was acidified indicating preferable blocking of the anion-exchange membrane. Indeed, after cleaning, its conductivity was 15 times lower. In the case of cation-exchange membrane, a decrease of conductivity was about 50%. This shows formation of precipitate inside pores of the pristine membranes.

## Discussion

Modification improves charge selectivity of the cation-exchange membrane (see Table 1), this is probably due to screening of pores with inorganic particles. The aggregated nanoparticles form secondary porous structures inside the membranes. Small pores between the nanoparticles as well as high surface charge density, which is realized in neutral media due to dissociation of phosphorus-containing functional groups [45], prevent transport of co-ions. At the same time, lower transport number of counter ions was found for the modified anion-exchange membrane. Indeed, HZD sorbs anions (An^−^) mainly in acidic media:

4 $$ \hbox{--} \mathrm{O}\mathrm{H}+\mathrm{H}\mathrm{A}\mathrm{n}\leftrightarrow \hbox{--} \mathrm{O}{{\mathrm{H}}_2}^{+}\mathrm{A}{\mathrm{n}}^{-} $$

and cations (Cat^+^) from alkaline solutions:

5 $$ \hbox{--} {\mathrm{O}}^{-}{\mathrm{H}}^{+}+\mathrm{C}\mathrm{a}{\mathrm{t}}^{+}\leftrightarrow \hbox{--} {\mathrm{O}}^{-}\mathrm{C}\mathrm{a}{\mathrm{t}}^{+}+{\mathrm{H}}^{+}. $$

Normally, isoelectric point of HZD is reached in neutral media: under these conditions cation- and anion-exchange capacities are equal. Thus, the HZD aggregates permit both counter (Cl^−^) and co- (Na^+^) ions. However, the aggregates protect the ion-exchange materials against fouling with organics.

Thus, ZHP increases the transport number of counter-ions in the cation-exchange membrane. At the same time, HZD slightly deteriorates charge selectivity of the anion-exchange membrane. Improvement of anion transport is expected in acidic media. However, the possibility of glycerol desalination is realized even in neutral media.

## Conclusions

As shown, the nanoparticles are aggregated in ion exchangers during their treatment with glycerol. In order to reach stability of incorporated nanoparticles and functional properties of the materials, the modification procedure was performed in glycerol media. Under these conditions, both non-aggregated nanoparticles and their small aggregates (up to 300 nm) are formed. They are evidently located inside voids between gel regions and perform a function of a barrier against adsorption of organic impurities on hydrophobic fragments of hydrocarbonaceous chains. No sufficient influence of ZHP on semi-permittivity of the cation-exchange membrane was found in aqueous NaCl solutions. At the same time, HZD slightly deteriorates charge selectivity of the anion-exchange membranes in neutral media due to amphoteric properties of the modifier. They are evidently a barrier against not only additionally sorbed electrolyte but also adsorption of organics.

The composite membranes were applied to desalination of glycerol-water mixture containing organic additives (byproduct of biodiesel production). In opposite to pristine membranes, the composite materials were shown to demonstrate stability against fouling. It is possible to decrease the salt concentration in 100 times, organic additives remain in the desalinated solutions. Acceleration of the desalination process requires improvement of the electrodialysis stack. Due to the problem of limiting current, deeper desalination can be carried out using ion exchange. Organic-inorganic ion exchangers modified in non-aqueous media could be probably used for this purpose.
